# Nephroprotective effects of GLP-1 receptor agonists in patients receiving lithium treatment: a multinational cohort study using the TriNetX network

**DOI:** 10.1017/S1092852926101023

**Published:** 2026-06-09

**Authors:** Roger S. McIntyre, Yanli Zhang-James, Joseph F. Goldberg, Angela T.H. Kwan

**Affiliations:** 1 Department of Psychiatry, University of Toronto, Canada; 2 Department of Psychiatry and Behavioral Sciences, SUNY Upstate Medical University Norton College of Medicine, USA; 3 Department of Psychiatry, Icahn School of Medicine at Mount Sinai, USA; 4 Brain and Cognition Discovery Foundation, Canada; 5 University of Toronto, Canada

**Keywords:** GLP-1 receptor agonists, lithium therapy, nephrotoxicity, renal outcomes, chronic kidney disease, acute kidney injury, type 2 diabetes mellitus, real-world evidence, propensity score matching, multinational cohort

## Abstract

**Background:**

Use of GLP-1 receptor agonists (GLP-1 RAs) is an established therapy that delays progression of chronic kidney disease in type 2 diabetes mellitus (T2DM), but their impact on lithium-associated nephrotoxicity is unclear. We evaluated whether GLP-1 RA use is associated with reduced renal risk in adults receiving long-term lithium therapy.

**Methods:**

We conducted a retrospective, propensity score-matched cohort study using TriNetX electronic health records (>127 million patients across 109 healthcare organizations). Adults (≥18 years) with T2DM and lithium exposure within 2 years prior to index were included. The primary outcome was renal nephrotoxicity, defined as a composite of ICD-10 and CPT-coded renal disease. Incidence and time-to-event outcomes were assessed using Kaplan–Meier curves and Cox proportional hazards models.

**Results:**

In a 24-month analysis of 452 matched patient pairs, GLP-1 RA initiation during lithium therapy was associated with a marked reduction in renal events versus lithium alone (23.5% vs 44.7%), corresponding to a risk difference of −21.2% (95% CI -36.9 to −5.5) and a risk ratio of 0.53 (95% CI 0.30–0.91; p = 0.013). Event-free survival was higher (62.5% vs 43.4%; log-rank p = 0.0115), with a hazard ratio of 0.45 (95% CI 0.23–0.85; p = 0.0089). This represents a 47% relative risk reduction and a number needed to treat (NNT) of 5 over 24 months.

**Conclusion:**

These findings support further mechanistic and prospective studies of GLP-1 RAs as a renoprotective strategy for patients receiving lithium treatment.

## Introduction

Lithium remains one of the most effective treatments for acute mania and long-term relapse prevention in bipolar I disorder, with benefits that extend beyond mood stabilization to include reduced suicide risk and possible neuroprotective effects.^1^ ^–^^3^ A major safety concern that requires regular laboratory monitoring and a common reason for both noninitiation and early discontinuation of lithium therapy is lithium-associated nephrotoxicity. Precisely estimating the frequency of lithium-associated chronic kidney disease in persons with long-term lithium exposure has been difficult because of multiple confounding factors that affect the risk estimate in the mood disorder population (e.g., type 2 diabetes mellitus [T2DM]).^4^ ^,^^5^ Nonetheless, the estimated population prevalence of chronic kidney disease (CKD) stage 3 or greater is 6% to 7%; in persons with T2DM, it is approximately 30% to 40%, while in persons with long-term lithium exposure, estimates have ranged from 27% to 32%.^6^ ^–^^8^ No intervention has been established as effective in the treatment or prevention of lithium-associated nephrotoxicity. Discontinuation of lithium in this context increases risk for the person with lived experience of bipolar disorder, and it has not necessarily been established that switching to an anticonvulsant (some of which are also associated with nephrotoxicity) consistently results in a reduction in chronic kidney disease progression.^4^ ^,^^9^ Nephrotoxicity due to lithium treatment has contributed to the reduction in use of lithium in individuals with bipolar disorder despite its longstanding position as the most effective maintenance treatment.^10^ ^,^^11^

GLP-1 receptor agonists (GLP-1 RAs) produce substantial glucose- and weight-lowering effects and are now widely used in the management of metabolic disease. GLP-1 receptor agonists also lower cardiovascular mortality in individuals with obesity; each of the aforementioned comorbidities are known to differentially affect persons living with depression or bipolar disorder.^12^ ^,^^13^ Pertinent to this analysis, GLP-1 RAs such as semaglutide are also FDA approved to reduce the risk of worsening kidney disease and progression to kidney failure in adults with T2DM and chronic kidney disease.^14^ ^,^^15^

Mechanisms hypothesized as mediators of the renal protective effects of GLP-1 RAs in T2DM overlap with mechanistic pathways implicated in lithium-associated nephrotoxicity.^16^ For example, long-term lithium exposure alters intracellular signaling, influences cellular proliferation, promotes extracellular matrix deposition and fibrosis, and activates oxidative and inflammatory cascades.^17^ Further impetus to consider GLP-1 RAs as potential treatments and/or prevention strategies for persons receiving lithium is a separate line of evidence which has preliminarily reported the nephroprotective effects of GLP-1 RAs in the context of exposure to other nephrotoxic agents (eg, cisplatin, tacrolimus).^18^ ^,^^19^ Whether GLP-1 RAs confer similar protection against lithium-associated nephrotoxicity has not been thoroughly evaluated in clinical populations.^20^ Herein, we examined whether GLP-1 RA exposure is associated with a reduction in a composite measure of lithium-related nephrotoxicity.

## Methods

### Study design and data source

We conducted a retrospective cohort study using the TriNetX Research Network, a federated cloud-based analytics platform that aggregates deidentified electronic health records from participating health systems worldwide.^21^ TriNetX harmonizes demographic, diagnostic, procedural, medication, and laboratory data using the Observational Medical Outcomes Partnership (OMOP) common data model. All analyses were executed within the secure TriNetX Live environment, and no identifiable information was accessed. Small cell counts (n ≤ 10) were automatically rounded by TriNetX to comply with privacy rules. Institutional Review Board approval was obtained at the SUNY Upstate Medical University (IRB #2302956–2).

### Study population

Eligible participants were adults aged 18 years or older with T2DM and at least one recorded lithium prescription. For each analytic window, individuals were required to have at least one lithium prescription during the 2 years preceding the index date of GLP-1 RA initiation and to have no GLP-1 RA exposure during that same period. Exclusion criteria included prior renal transplantation, active pregnancy, and missing demographic variables. The 2-year interval preceding the index date was selected as the primary exposure window to ensure that participants had sustained, multiyear lithium treatment before GLP-1 RA initiation.

GLP-1 receptor agonist exposure was defined using a prespecified concept set that included semaglutide, liraglutide, dulaglutide, exenatide, lixisenatide, and tirzepatide. Comparator patients were individuals treated with lithium who had no GLP-1 RA prescriptions during the 2 years before cohort entry and who remained unexposed to GLP-1 RAs during follow-up. For patients with GLP-1 RA exposure, the index date was defined as the date of the first qualifying GLP-1 RA prescription. For comparator patients, the index date was defined as the first qualifying lithium prescription. In both groups, exposure was required to occur within the same relative time window (from 24 months prior to the index date up to the index date). Each analytic window was constructed independently, and identical timing rules were applied to both groups.

### Comparator populations

It has been established that GLP-1 RAs are nephroprotective in persons living with T2DM. Consequently, to explore whether GLP-1 RAs may confer protection against lithium-associated nephrotoxicity beyond their established renal benefits in T2DM, we conducted a sensitivity analysis comparing individuals with T2DM prescribed both a GLP-1 RA and lithium with those prescribed a GLP-1 RA alone. For both groups, the index date was defined as the first GLP-1 RA prescription, and kidney outcomes occurring before this date, including those arising after lithium initiation but prior to GLP-1 RA initiation, were excluded.

### Outcome measures and primary efficacy endpoint

The primary outcome was a composite renal endpoint defined using International Classification of Diseases, Tenth Revision (ICD-10), and Current Procedural Terminology (CPT) codes to capture clinically significant renal events across acute and chronic kidney disease. Components included acute kidney injury (N17.x; N17.0-N17.2, N17.8-N17.9), chronic kidney disease (N18.3-N18.6, N18.9), kidney failure (N19), end-stage renal disease (N18.6), dialysis-related outcomes (Z99.2; CPT 90935, 90 937), transplant-related outcomes (T86.10-T86.19, Z94.0), and intrinsic renal disorders (N05, N05.8, N12, N28). For interpretability, components were grouped into clinically meaningful categories: acute kidney injury, chronic kidney disease (N18.3-N18.6, N18.9), kidney failure, dialysis-related outcomes, transplant-related outcomes, and other intrinsic renal disorders.

The primary efficacy endpoint was the rate of composite renal events among persons with T2DM prescribed both lithium and a GLP-1 receptor agonist compared with those prescribed lithium without a GLP-1 receptor agonist. A prespecified sensitivity analysis compared persons receiving both a GLP-1 receptor agonist and lithium with those receiving a GLP-1 receptor agonist alone. To address heterogeneity within the composite endpoint, an additional sensitivity analysis excluded acute kidney injury-related diagnoses (ICD-10 N17.x), with the remaining composite representing non-AKI renal outcomes.

A negative control outcome consisting of benign dermatologic conditions unrelated to renal injury was also assessed, including melanocytic nevi (D22.0-D22.9), benign cutaneous neoplasms (D23.0-D23.9), and seborrheic keratoses (L82.0-L82.1). These conditions were selected a priori, as they share no known pathophysiological overlap with renal injury or GLP-1 receptor agonist mechanisms, thereby serving as a methodological control to assess for residual confounding or systematic bias.

Follow-up extended from the index date until the earliest of a primary outcome event, negative control event, death, loss to follow-up, or completion of the 24-month follow-up period.

### Covariates

Baseline covariates included age, sex, hypertension, disorders of lipoprotein metabolism, overweight and obesity, and ischemic heart disease. All covariates were identified from diagnoses recorded during the defined exposure interval preceding the index date.

### Propensity score matching

Propensity scores were estimated using logistic regression including all baseline covariates. Exposed and comparator patients were matched one to one using nearest-neighbor matching without replacement and a caliper of 0.1 of the pooled standard deviation of the logit of the propensity score. Covariate balance was assessed using standardized differences, with values less than 0.1 indicating adequate balance. Matching was performed independently for each analytic window and for each of the four sensitivity comparisons.

### Statistical analysis

Event counts, risks, risk differences, and risk ratios were calculated from the matched cohorts. Kaplan–Meier methods and log-rank tests were used for time-to-event analysis. Cox proportional hazards models generated hazard ratios with 95% confidence intervals. All statistical procedures were conducted using the validated TriNetX analytics engine. A two-sided alpha of 0.05 indicated statistical significance.

## Results

### Study population

**Figure 1.**
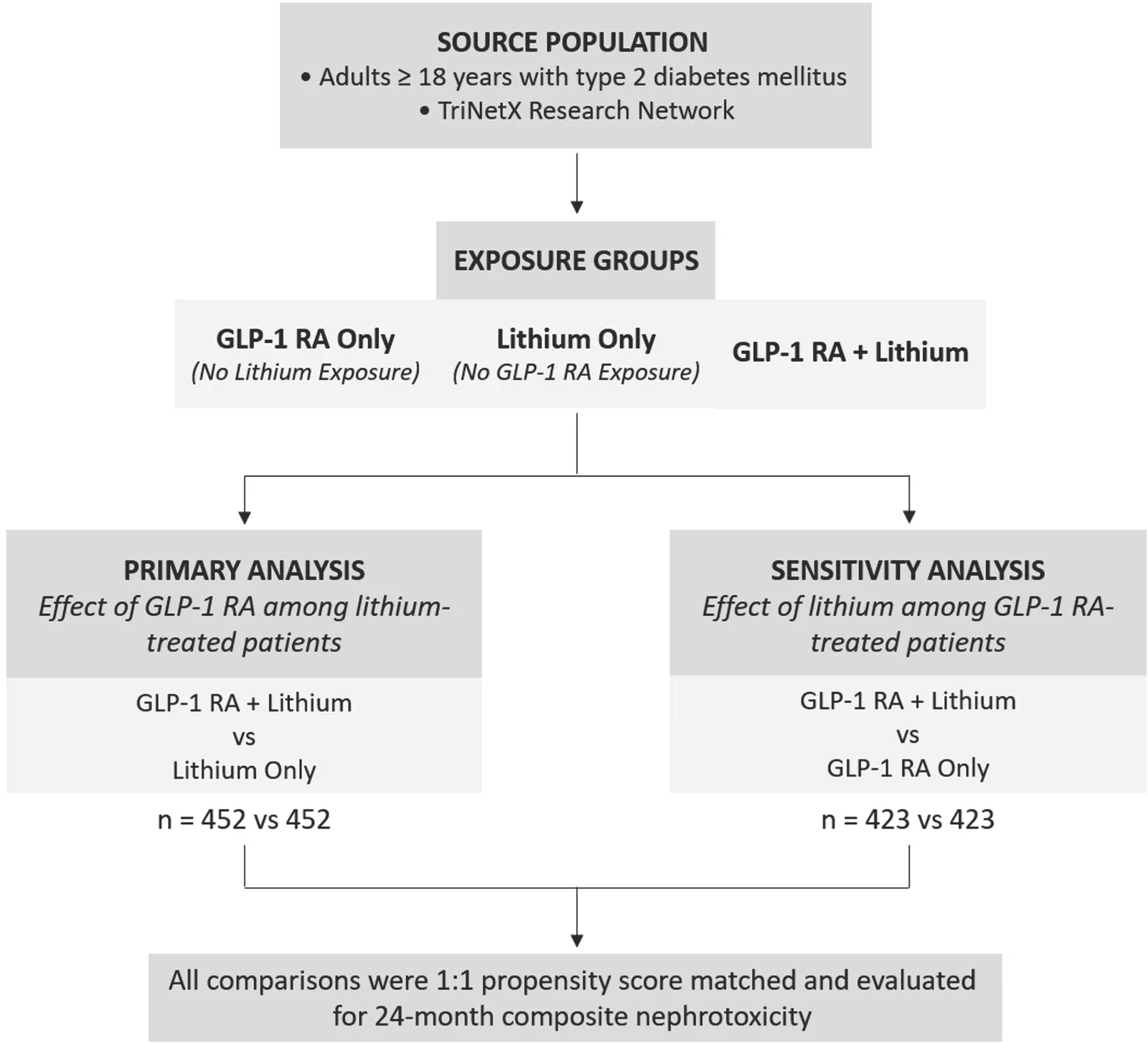
Cohort derivation and analytic design: flowchart illustrating patient selection and construction of matched comparator groups to disentangle the independent effects of GLP-1 receptor agonists and lithium. Figure 1. long description.

A total of 452 matched patient pairs were included in the comparison of patients with T2DM treated with lithium plus a GLP-1 RA versus lithium alone (Figure 1). Propensity-score matching (PSM) using the TriNetX 1:1 nearest-neighbor algorithm achieved good overall covariate balance, with most standardized differences <0.05 for demographic and cardiometabolic characteristics.

The cohorts were closely aligned in age (49.6 ± 13.6 vs 49.3 ± 14.2 years; standardized difference = 0.024) and age at index (48.6 ± 13.6 vs 48.0 ± 14.2 years; standardized difference = 0.048). Sex distribution was also well balanced (female: 68.6% vs 69.7%; standardized difference = 0.024) (Table 1). Cardiometabolic comorbidities were generally well balanced after matching, including T2DM (31.9% vs 34.5%), overweight/obesity (23.5% vs 21.0%), hypertension (11.3% vs 10.2%), dyslipidemia (11.7% vs 10.6%), and ischemic heart disease (2.2% vs 2.2%).

**Table 1.** Baseline Clinical and Demographic Profile of Adults with Type 2 Diabetes Mellitus and Recent Lithium Exposure: Comparison of Patients Treated with GLP-1 Receptor Agonists versus Lithium, Controls Without GLP-1 Exposure, Before and After Propensity Score Matching Table 1. long description.

	Before propensity score matching			After propensity score matching		
	GLP-1 RA-exposed lithium cohort	GLP-1 RA-naïve lithium cohort	SMD (Standardized Mean Difference)	GLP-1 RA-exposed lithium cohort	GLP-1 RA-naïve lithium cohort	SMD
Total number	457	6111	–	452	452	–
Age at the index event, years, mean ± s.d.	48.6 ± 13.6	52.8 ± 15.4	0.29	48.6 ± 13.6	48 ± 14.2	0.048
Sex (%)						
Female	68.9%	54.3%	0.31	68.6%	69.7%	0.024
Male	31.1%	45.61%	0.30	31.42%	30.3%	0.024
Diabetes mellitus	32.2%	23.52%	0.19	31.9%	34.5%	0.056
Overweight and obesity	24.3%	7.5%	0.47	23.5%	21.0%	0.059
Disorders of lipoprotein metabolism and other lipidemias	11.6%	13.0%	0.043	11.7%	10.62%	0.035
Hypertensive diseases	11.2%	17.2%	0.17	11.3%	10.2%	0.036
Ischaemic heart diseases	2.2%	4.6%	0.13	2.2%	2.2%	< 0.0001
Acute Kidney Failure	2.2%	3.2%	0.060	2.2%	2.2%	< 0.0001
Chronic Kidney Disease, Stage 3 (Moderate)	2.2%	1.5%	0.055	2.2%	2.2%	< 0.0001
Chronic kidney disease, stage 3 (Unspecified)	2.2%	1.0%	0.097	2.2%	0%	0.2
Chronic kidney disease, stage 4	0%	0.3%	0.075	0%	2.2%	0.2
Chronic kidney disease, stage 5	0%	0.2%	0.057	0%	0%	–
Tubulointerstitial nephritis	0%	0.2%	0.060	0%	0%	–

Baseline renal covariates were also included in the matching process; however, because several renal diagnoses were uncommon, some residual imbalance persisted for low-frequency codes. Notably, baseline prevalence of chronic kidney disease and acute kidney injury was low (each approximately 2% to 3% of the cohort), limiting the impact of these imbalances on overall comparability.

Follow-up time was comparable between groups (mean 265.7 vs 331.5 days; median 253 vs 314 days; interquartile range [IQR] 357 vs 433 days). Each sensitivity comparison used an independently matched cohort drawn from the broader diabetes population.

### Primary analysis: renal outcomes in patients with T2DM receiving lithium with vs without GLP-1 receptor agonists

**Figure 2.**
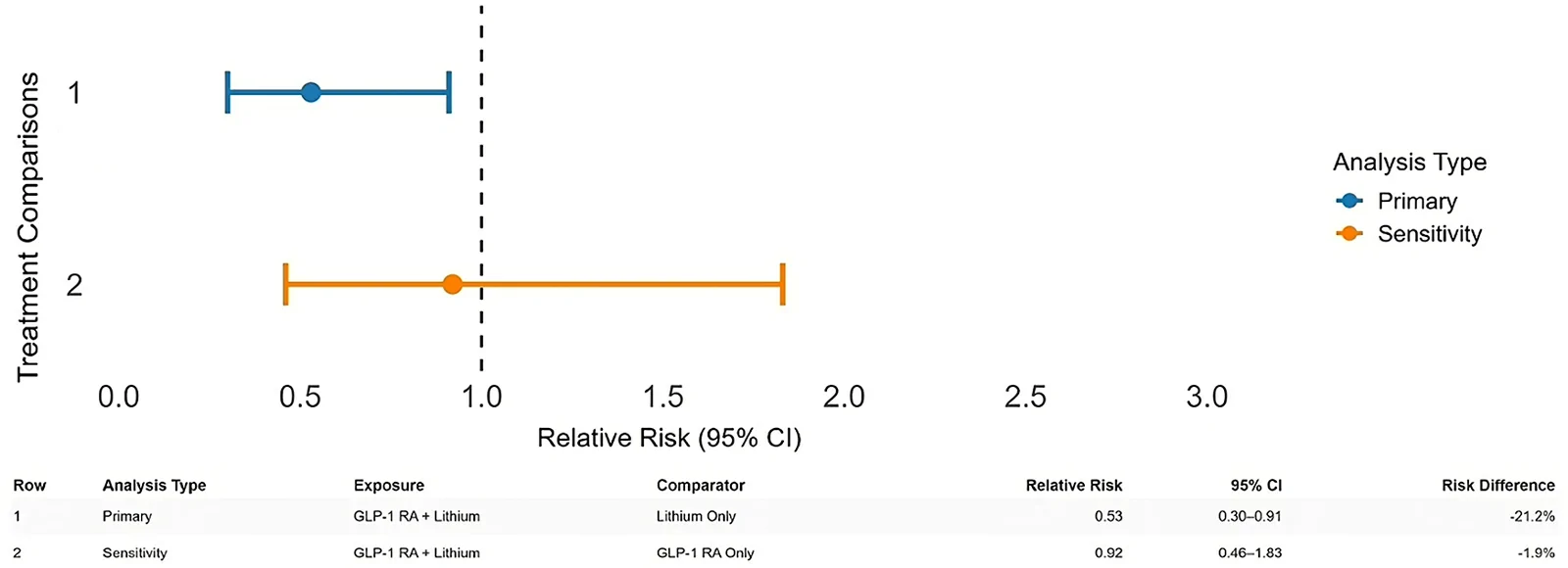
Comparative risk of composite nephrotoxicity across primary and sensitivity analyses in matched cohorts of adults with type 2 diabetes. Forest plot of relative risks (95% confidence intervals) derived from independent 1:1 propensity score-matched cohorts. The primary analysis (blue) contrasts lithium-exposed adults initiating GLP-1 receptor agonists with matched lithium-treated individuals not exposed to GLP-1 receptor agonists. The sensitivity analysis (orange) contrasts combined GLP-1 receptor agonist plus lithium therapy with matched GLP-1 receptor agonist monotherapy to further isolate lithium-associated risk.

For participants who were prescribed lithium within at least 24 months prior to a GLP-1 RA prescription, a lower incidence of renal outcomes compared with those treated with lithium alone was observed (23.5% vs 44.7%; risk difference 21.2% [95% CI -36.9 to −5.5]; risk ratio 0.53 [95% CI 0.30–0.91]; p = 0.013) (Figure 2). Kaplan–Meier curves revealed consistent time-to-event findings, with 24-month event-free survival of 62.5% versus 43.4% (log-rank p = 0.0115; hazard ratio 0.45 [95% CI 0.23–0.85]; p = 0.0089). Overall, these findings indicate that adding a GLP-1 RA to chronic lithium therapy corresponds to a 21.2% absolute and 47% relative reduction in renal events, reflecting a clinically meaningful improvement over lithium therapy without a GLP-1 RA. This corresponds to an estimated number needed to treat (NNT) of 5 over a 24-month period.

To further assess the contribution of acute kidney injury to the composite outcome, a sensitivity analysis excluding acute kidney injury-related diagnoses (ICD-10 N17.x) was performed over a 24-month follow-up period. In this analysis, GLP-1 receptor agonist use remained associated with a significantly lower risk of renal events compared to controls (risk ratio 0.498, 95% CI 0.28–0.88; p = 0.0094), reinforcing that the observed association is unlikely to be driven solely by acute kidney injury and may extend to broader, nonacute renal disease processes. Within the composite endpoint, events were predominantly driven by acute kidney injury-related diagnoses, with smaller contributions from non-AKI renal conditions.

### Sensitivity analysis: renal outcomes in patients with T2DM receiving GLP-1 receptor agonists with vs without lithium

Among 423 matched patients per group, GLP-1 RA plus lithium showed a similar 24-month renal event rate compared with GLP-1 RA therapy alone (21.6% vs 23.4%; risk difference 1.9% [95% CI -17.2 to 13.5]; risk ratio 0.92 [95% CI 0.46–1.83]; p = 0.8119) (Supplementary Table S1). This difference was not statistically significant, and confidence intervals crossed the null. Kaplan–Meier curves showed no meaningful separation, with 24-month event-free survival of 65.3% versus 67.7% (log-rank p = 0.8060; hazard ratio 0.91 [95% CI 0.42–1.98]; p = 0.1420). Overall, these findings indicate that among GLP-1 RA–treated patients, concurrent lithium exposure was not associated with a statistically significant difference in renal event risk over 24 months.

### Negative control analysis

Among 462 matched patient pairs, benign dermatologic outcomes occurred at similar rates between exposure groups (3.9 percent versus 2.2 percent; risk difference + 1.7 percent [95 percent CI -0.48 to 3.94]; risk ratio 1.80 [95 percent CI 0.84–3.86]; p = 0.13). Kaplan–Meier curves showed no meaningful separation, with 24-month event-free survival remaining comparable across groups.

## Discussion

In this large multiinstitutional cohort of adults with T2DM receiving lithium therapy, initiation of a GLP-1 receptor agonist was associated with an approximately 47% relative reduction in renal risk among individuals with lithium exposure within the preceding 2 years. Findings from sensitivity analyses were consistent with the primary results, as GLP-1 receptor agonist use in patients receiving lithium was associated with a risk of renal events not significantly different from those receiving GLP-1 receptor agonists alone. The persistence of this association after exclusion of acute kidney injury-related events suggests that the observed effect is not solely driven by acute renal injury and may extend to broader, non-acute renal disease processes. Importantly, this corresponded to an absolute risk reduction of 21.2%, yielding an estimated NNT of 5 over a 24-month period, underscoring the clinical relevance of the observed association.

The nephroprotective effects of GLP-1 RAs in T2DM are not fully accounted for by improvement in metabolic parameters alone. For example, GLP-1 RAs are reported to lower albuminuria by 20 to 40% and slow eGFR decline across multiple independent studies and meta-analyses.^14^ ^,^^22^ ^-^^27^ Experimental and translational studies have reported GLP-1 receptor signaling in renal vascular and tubular cells wherein they suppress inflammatory and oxidative pathways, reducing fibrotic injury.^28^ ^,^^29^ GLP-1 receptor agonists enhance tubular and microvascular resilience via multiple pathways including enhanced renal perfusion, reduced hyperfiltration, and natriuresis with associated reduced blood-pressure. In addition, GLP-1 RAs also enhance lipid disposition, reducing measures of lipotoxic stress, which may also be nephroprotective.^16^ ^,^^25^ ^,^^28^ Results from the FLOW study reported that semaglutide reduced major kidney events by 24% and reduced annual eGFR loss; results that have been reinforced by separate meta-analyses that have documented 16 to 19% reductions in kidney failure and related outcomes.^14^ ^,^^23^ ^–^^25^ In contrast, non-GLP-1 antidiabetic therapies provide glycemic control and produce modest renal benefit.^27^ ^,^^30^

Interpreting our results calls attention to the distinct pathophysiology of lithium-induced nephropathy, which overlap with but is not identical to mechanisms implicated in diabetic kidney disease.^31^ ^–^^33^ For example, chronic lithium therapy results in a predominantly tubulointerstitial pattern of injury with relative glomerular sparing.^31^ ^,^^34^ Long-term lithium exposure have been reported to result in chronic tubulointerstitial nephritis, tubular atrophy, interstitial fibrosis, and numerous 1 to 2 millimeter microcysts in distal nephron segments.^31^ ^,^^32^ Renal microcysts are a defining feature of lithium-induced nephrotoxicity, alongside biopsy and autopsy study reports of patchy scarring with tubular dilatation affecting up to 62% of long-term lithium users.^32^ ^,^^35^ Histopathologic data suggest that significant injury typically appears after long-term exposure and would not be expected with acute treatment alone.^32^ ^,^^36^ This distinction is particularly relevant given that lithium-associated nephropathy is primarily a chronic tubulointerstitial process, whereas acute kidney injury events may reflect transient or reversible insults.

### Strengths and limitations

The strength of our analysis is that to our knowledge, this is the largest cohort of individuals with lithium exposure within the preceding 24 months and were also prescribed a GLP-1 RA, and the overarching research question is whether GLP-1 RAs reduce composite nephrotoxicity risk in this context.^20^ Use of the TriNetX platform and PSM enhances confidence in the findings by balancing key covariates, reducing bias inherent to spontaneous reporting strengthening confidence in our findings. As most individuals receiving lithium have mood disorders, this study also provides the first population-level assessment of renal outcomes with GLP-1 RA therapy in a psychiatric population.

The limitations arise from the retrospective observational design. Although we used PSM, we cannot rule out the possibility of residual confounding. Unmeasured factors relevant to renal vulnerability, including cumulative lithium exposure, serum levels, treatment duration, concomitant nephrotoxins, and baseline tubular function, may also influence the observed effect estimates.^37^ In addition, we categorized GLP-1 RAs as a single therapeutic class despite their pharmacological heterogeneity, and our use of a composite renal outcome precludes conclusions about specific renal disease subtypes that may be differentially affected. Although stratified analyses by individual GLP-1 receptor agonists were explored, event counts within subgroups were limited following PSM, precluding reliable estimation of agent-specific effects. Accordingly, the present findings should be interpreted as reflecting a class-level association rather than agent-specific effects, and future studies are warranted to evaluate potential differences between individual GLP-1 receptor agonists. We also recognize that the 24-month lithium exposure window may not fully capture cumulative lifetime exposure, which is more relevant to chronic lithium-associated nephropathy. Accordingly, our findings should be interpreted as reflecting renal risk associated with recent or ongoing lithium exposure rather than long-term cumulative toxicity. Furthermore, because the composite endpoint includes both acute and non-acute renal diagnoses, the results should be interpreted as reflecting overall renal risk rather than a specific renal disease mechanism. Moreover, we cannot precisely estimate how long people have been receiving lithium or fidelity to taking either lithium or GLP-1 RA. In addition, we cannot completely exclude the possibility of immortal time bias, as nephrotoxicity related to prior lithium exposure may have predated the index exposure date but not have been fully captured in structured electronic health record data.

Moreover, we compared lithium-exposed persons with GLP-1 RA exposure to those lithium-exposed without GLP-1 RA exposure, which may introduce the potential for forward-looking exposure classification and related bias. This issue is critical to interpretation as treatment status in observational data may be influenced by clinical factors and treatment decisions occurring after cohort entry that are not fully captured in the dataset. Furthermore, because outcomes and exposures were identified using coded electronic health record data, misclassification of the timing or occurrence of renal events cannot be excluded, and some renal outcomes may have represented prevalent rather than strictly incident events. Residual confounding related to treatment selection and differences in underlying clinical risk between exposure groups may also persist despite analytic adjustment, and the negative control outcome may not fully share the same confounding structure as renal injury. These findings should be interpreted as reflecting an association rather than causation. Furthermore, given the use of a composite renal endpoint, the results do not permit estimation of risk reduction for individual kidney-specific outcomes.

Our methodology does not exclude the possibility that the GLP-1 RA mediated nephroprotective effects are not specific to lithium but represent a general renal protective effect. It is notable however that the preliminary evidence does suggest that GLP-1 RAs potentially mitigate renal toxicity from well-known nephrotoxic agents (eg, cisplatin, tacrolimus). These observations suggest that GLP-1 RAs target molecular and cellular systems relevant to nephrotoxicity from multiple causes including iatrogenic.^18^ ^,^^19^ ^,^^38^ ^–^^40^.

## Conclusion

Results from our study provide preliminary support for the hypothesis that GLP-1 RAs may have protective effects against lithium-associated nephrotoxicity. We did not address the pharmacokinetic interactions between lithium and GLP-1 RA exposure, which has been inadequately characterized so far in the literature. Case reports of lithium-related toxicity in persons exposed to incretin agonists have appeared, which have been attributed to reductions in volume status due to dehydration, polyuria, and/or vomiting, and/or effects on absorption due to delayed gastric emptying and/or displacement of lithium from the muscle compartment due to muscle wasting.^41^ ^,^^42^ Consequently, clinicians coprescribing lithium with GLP-1 receptor agonists should implement close monitoring of serum lithium levels, particularly during initiation and dose escalation, and provide proactive patient education regarding hydration and early symptoms of lithium toxicity. These precautions may help mitigate potential pharmacokinetic fluctuations and reduce the risk of adverse events in this clinically vulnerable population. Replication, as well as observational, clinical, and mechanistic studies are warranted to further strengthen confidence in the findings. More specifically, whether GLP-1 receptor agonists alleviate histopathologic evidence of lithium-associated nephrotoxicity should be a priority research consideration.

## Supporting information

10.1017/S1092852926101023.sm001 McIntyre et al. supplementary material McIntyre et al. supplementary material

## Supplementary material

The supplementary material for this article can be found at http://doi.org/10.1017/S1092852926101023.

## Data sharing statement

The individual-level electronic health record data used in this study are not publicly available because access is restricted by contractual agreements and institutional data use policies of the TriNetX research network. Researchers may obtain access to the data through TriNetX subject to institutional approval and a data use agreement. Aggregate results are provided in the manuscript are available from the authors upon reasonable request.

## Long descriptions

### Figure 1. Long description

The flowchart begins at the top with the SOURCE POPULATION, defined as Adults greater than or equal to 18 years with type 2 diabetes mellitus from the TriNetX Research Network.

An arrow points down to EXPOSURE GROUPS, which are divided into three categories:

1. G L P-1 R A Only (No Lithium Exposure).

2. Lithium Only (No G L P-1 R A Exposure).

3. G L P-1 R A plus Lithium.

From these groups, arrows branch into two parallel analysis paths:

On the left, PRIMARY ANALYSIS: Effect of G L P-1 R A among lithium-treated patients. This compares G L P-1 R A plus Lithium versus Lithium Only, with a sample size of n equals 452 versus 452.

On the right, SENSITIVITY ANALYSIS: Effect of lithium among G L P-1 R A-treated patients. This compares G L P-1 R A plus Lithium versus G L P-1 R A Only, with a sample size of n equals 423 versus 423.

Both paths converge at the bottom to a final box stating: All comparisons were 1 to 1 propensity score matched and evaluated for 24-month composite nephrotoxicity.

Navigate back to Figure 1..

### Table 1. Long description

The table is divided into two primary sections: Before propensity score matching and After propensity score matching. Each section contains three columns: G L P-1 R A-exposed lithium cohort, G L P-1 R A-naïve lithium cohort, and S M D (Standardized Mean Difference).

Key data points before matching:

* Total number: 457 (exposed) vs 6,111 (naïve).

* Mean age: 48.6 plus or minus 13.6 vs 52.8 plus or minus 15.4 (S M D 0.29).

* Female sex: 68.9% vs 54.3% (S M D 0.31).

* Overweight and obesity: 24.3% vs 7.5% (S M D 0.47).

Key data points after matching:

* Total number: 452 (exposed) vs 452 (naïve).

* Mean age: 48.6 plus or minus 13.6 vs 48 plus or minus 14.2 (S M D 0.048).

* Female sex: 68.6% vs 69.7% (S M D 0.024).

* Overweight and obesity: 23.5% vs 21.0% (S M D 0.059).

* Ischaemic heart diseases: 2.2% vs 2.2% (S M D less than 0.0001).

* Acute Kidney Failure: 2.2% vs 2.2% (S M D less than 0.0001).

* Chronic Kidney Disease Stage 3 (Moderate): 2.2% vs 2.2% (S M D less than 0.0001).

* Chronic kidney disease stage 5 and Tubulointerstitial nephritis: Both cohorts show 0% after matching.

Navigate back to Table 1..
