# Supplementary material for: Nephroprotective effects of GLP-1 receptor agonists in patients receiving lithium treatment: a multinational cohort study using the TriNetX network
Source: CNS Spectr. 2026 Jun 9;31(1):e25. doi: 10.1017/S1092852926101023 (PMC13732603; doi:10.1017/S1092852926101023)
Supplement: Supplementary file 1 [file S1092852926101023sup001.docx]

**Supplementary Table**

**Table S1.** Baseline characteristics of patients with type 2 diabetes mellitus receiving GLP-1 receptor agonists with vs without lithium exposure, before and after propensity score matching

|  | **Before propensity score matching** | | |  | **After propensity score matching** | | |
| --- | --- | --- | --- | --- | --- | --- | --- |
|  | **GLP-1 RA With Lithium** | **GLP-1 RA Without Lithium** | **SMD** |  | **GLP-1 RA With Lithium** | **Lithium**  **GLP-1 RA Without Lithium** | **SMD** |
| Total number | 424 | 211,620 | - |  | 423 | 423 | - |
| Age at the index event, years, mean ± s.d. | 48.8 ± 13.6 | 57.1 ± 13 | 0.62 |  | 48.8 ± 13.6 | 48.8 ± 13.6  49 | 0.0026 |
| Sex (%) |  |  |  |  |  |  |  |
| Female | 69.1% | 65.4% | 0.079 |  | 69.0% | 68.8% | 0.0051 |
| Male | 30.9% | 34.5% | 0.077 |  | 31.0% | 31.2% | 0.0051 |
| Diabetes Mellitus | 31.1% | 42.9% | 0.25 |  | 31.2% | 31.4% | 0.0051 |
| Overweight and Obesity | 23.3% | 22.6% | 0.019 |  | 23.2% | 23.2% | < 0.0001 |
| Disorders of Lipoprotein Metabolism and Other Lipidemias | 11.8% | 21.2% | 0.26 |  | 11.8% | 11.8% | < 0.0001 |
| Hypertensive Diseases | 11.3% | 24.2% | 0.34 |  | 11.3% | 11.1% | 0.0075 |
| Ischaemic Heart Diseases | 2.4% | 3.3% | 0.057 |  | 2.4% | 2.4% | < 0.0001 |
| Acute Kidney Failure | 2.4% | 0.1% | 0.20 |  | 2.4% | 0% | 0.22 |
| Chronic Kidney Disease, Stage 3 (Moderate) | 2.4% | 2.0% | 0.02 |  | 2.4% | 2.4% | < 0.0001 |
| Chronic Kidney Disease, Stage 3 (Unspecified) | 2.4% | 0.3% | 0.18 |  | 2.4% | 2.4% | < 0.0001 |
| Chronic Kidney Disease, Stage 4 | 0% | 0.3% | 0.083 |  | 0% | 0% | - |
| Chronic Kidney Disease, Stage 5 | 0% | 0.08% | 0.039 |  | 0% | 0% | - |
| Tubulointerstitial nephritis | 0% | 0.02% | 0.019 |  | 0% | 0% | - |
